# circ_0004904 regulates the trophoblast cell in preeclampsia via miR-19b-3p/ARRDC3 axis

**DOI:** 10.1515/med-2022-0546

**Published:** 2023-05-16

**Authors:** Chenyuan Cao, Jie Cui, Guiling Liu

**Affiliations:** Department of Obstetrics, The Affiliated Hospital of Hebei University, Baoding City, Hebei Province, 071000, China

**Keywords:** preeclampsia, circ_0004904, miR-19b-3p, ARRDC3

## Abstract

Circular RNAs have been demonstrated to act as vital participants in various diseases, including preeclampsia (PE). This study aimed to research the effects of circ_0004904 on PE. The contents of circ_0004904, microRNA-19b-3p (miR-19b-3p) and arrestin domain containing 3 (ARRDC3) were quantified by quantitative real-time PCR and western blot. The 3-(4,5-dimethylthiazol-2-yl)-2,5-diphenyltetrazolium bromide and 5-ethynyl-2′-deoxyuridine assays were enforced to assess cell proliferation. The transwell assay and flow cytometry were applied to detect the cell migration, invasion, and apoptosis. The liaison between miR-19b-3p and circ_0004904 or ARRDC3 was demonstrated by dual-luciferase reporter assay. Thereafter, circ_0004904 and ARRDC3 were augmented, and miR-19b-3p was restrained in PE. Circ_0004904 silencing contributed to cell proliferation, migration, and invasion, but restrained cell apoptosis in trophoblast cells. Further, miR-19b-3p was a target of circ_0004904, and miR-19b-3p could target ARRDC3. Additionally, circ_0004904 accelerated PE evolution via changing ARRDC3 level by binding to miR-19b-3p. In all, circ_0004904 encouraged PE progress via miR-19b-3p/ARRDC3 axis.

## 1 Introduction

Preeclampsia (PE) is a clinical condition characterized by hypertension, proteinuria, and edema, often occurring in the last trimester of pregnancy [1,2]. Its incidence is usually 3–7% of all pregnant women [3]. PE can be caused by many factors, such as high blood pressure, proteinuria, obesity, multiple pregnancies, and age at the time of conception [4]. If left untreated, PE can lead to maternal and newborn deaths [5]. At present, there are many doubts about the pathogenesis and regulatory mechanism of PE, and further research is needed.

Circular RNAs (circRNAs) are one of the endogenous RNAs with stable closed-loop structure, which have attracted extensive attention in disease regulation [6,7]. More importantly, some studies have found the abnormal expression of some circRNAs in the placenta of PE patients, which may be closely related to the pathogenesis of PE [8]. For instance, hsa_circ_0004904 and hsa_circ_0001855 were conspicuously intensified in PE, which might be the markers for PE forecasting [9]. However, the regulation mechanism of circ_0004904 in PE is still unclear, which will be the focus of this research. Moreover, hsa_circ_0036877 acted as a possible plasma biomarker for PE [10]. Besides, circHIPK3 participated in cell migration and tube formation in PE [11]. In addition, circ_0001438 took part in cell migration and apoptosis in PE [12]. In this work, we mainly studied the role of circ_0004904 in PE and its regulatory mechanism.

MicroRNAs (miRNAs) are diminutive noncoding RNAs and are concerned with numerous cell biological functions [13]. For example, miR-203a-3p and miR-548c-5p were essential participants in inflammatory response of PE [14,15]. Besides, miR-125a-5p repressed cell migration and proliferation in PE [16]. In addition, Sandrim et al. proved that microRNA-19b-3p (miR-19b-3p) expression was downregulated in PE [17]. But, the meaning and mechanism of miR-19b-3p in PE is not completely addressed.

Arrestin domain containing 3 (ARRDC3) is a member of the α-arrestin family [18]. Shen et al. reported that ARRDC3 contributed to YAP degradation, then accelerated colorectal cancer development [19]. Besides, ARRDC3 took part in the regulation of breast cancer [20]. Furthermore, ARRDC3 level was elevated and upregulated ARRDC3 lessened tube formation in PE [21]. Whereas, the regulation mechanism of ARRDC3 in PE is still unclear.

Here our results displayed that circ_0004904 was amplified in PE tissues and cells, and circ_0004904 silencing contributed to cell proliferation, migration, and invasion, but restrained cell apoptosis in trophoblast cells. Hence, we designed to recognize whether circ_0004904 could adjust PE growth via miR-19b-3p/ARRDC3 axis.

## 2 Materials and methods

### 2.1 Samples, cell culture, and transfection

The research was supervised by the Affiliated Hospital of Hebei University. 41 pairs PE placental tissues and normal placental tissues were gathered from The Affiliated Hospital of Hebei University. All volunteers signed the informed consent, and the present study was authorized by the Ethics Committee of The Affiliated Hospital of Hebei University.

Human trophoblast cell line (HTR-8/SVneo) was obtained from American Type Culture Collection (ATCC, Manassas, VA, USA). The detailed process of cell culture was as the process reported by Gai et al. [22].

The si-circ_0004904, si-NC, miR-19b-3p mimic, miR-497-5p inhibitor, and controls, the pcDNA3.0-ARRDC3 and pcDNA3.0-NC, were from Sangon (Shanghai, China). The transfection was enforced by Lipofectamine 3000 (Solarbio, Beijing, China).

### 2.2 Quantitative real-time PCR (qRT-PCR) and RNA degradation assay

The TRIzol (Takara, Dalian, China) was utilized for RNA extraction. Then, the RNA was used for reverse transcription. After that, the qRT-PCR was enforced in line with the previous report [22]. GAPDH was utilized to standardize circ_0004904 and ARRDC3 levels, and U6 was employed to normalize miR-19b-3p content. All statistics were figured by 2^−ΔΔCt^ method. RNase R (Solarbio) and Act D were applied to manifest the circular form of circ_0004904. The primers are listed in Table 1.

**Table 1:** Primers for qRT-PCR

Name		Primers for qRT-PCR (5′−3′)
circ_0004904	Forward	GAGGACCCACAGGCATGAAT
	Reverse	AGCGTGGAATATCAAATGCTCC
ARRDC3	Forward	GGCACAAAAAGGGAGCGAAG
	Reverse	TGCAAATTCCCCTGAACGGA
miR-19b-3p	Forward	GCCGAGTGTGCAAATCCATGCT
	Reverse	CTCAACTGGTGTCGTGGA
GAPDH	Forward	TCCCATCACCATCTTCCAGG
	Reverse	GATGACCCTTTTGGCTCCC
U6	Forward	CTCGCTTCGGCAGCACATATACT
	Reverse	ACGCTTCACGAATTTGCGTGTC
18S rRNA	Forward	CAGCCACCCGAGATTGAGCA
	Reverse	TAGTAGCGACGGGCGGTGTG
POLE2	Forward	TGCCTCTGCATAAACCCTGG
	Reverse	TCTCAAAAGCCTTGAAGTTTGCT

### 2.3 Western blot

The technique of western blot was conducted as reported in a previous study [23]. The antibodies used for western blot were as follows: anti-ARRDC3 (ab64817; 1:500; Abcam, Cambridge, MA, USA), anti-cyclin E (ab33911; 1:1,000; Abcam), anti-vimentin (ab92547; 1:1,000; Abcam), anti-MMP2 (ab92536; 1:1,000; Abcam), anti-Bcl-2 (ab32124; 1:500; Abcam), anti-Bax (ab32503; 1:1,000; Abcam), and anti-GAPDH (ab8245; 1:5,000; Abcam).

### 2.4 Cell proliferation assay

HTR-8/SVneo cells (1 × 10^5^/well) were loaded on 96-well plates. The MTT (Sigma) solution was employed as reported previously by Chen et al. [24]. Finally, the absorbance at 490 nm was observed with a microplate reader. Cell proliferation was also measured by Cell-Light™ Edu Kit (RiboBio, China) as stated in the directions.

### 2.5 Transwell assay

After transfection, the HTR-8/SVneo cells were supplemented into the above chamber of the transwell (Corning Inc., Corning, NY, USA) pre-coated with or without Matrigel (Corning) to assess the ability of cell invasion and migration, respectively. Then, the 200 µL of complete medium was added into the lower chamber. After 48 h, the moved cells were stained and calculated with a microscope.

### 2.6 Flow cytometry assay

HTR-8/SVneo cells were loaded on 6-well plates. The apoptotic cells were quantified via Annexin V-FITC Apoptosis Detection Kit (Sigma) and investigated using a flow cytometer.

### 2.7 ELISA assay

The activity of Caspase-3 was measured through human active Caspase-3 ELISA kit (ab181418; Abcam) in accordance with the specification.

### 2.8 Dual-luciferase reporter assay

The test was carried out based on the procedure reported by Gai et al. [22]*.* The connection between miR-19b-3p and circ_0004904 or ARRDC3 was predicted by starBase (http://starbase.sysu.edu.cn). Then, the circ_0004904 and ARRDC3 wild and mutant vectors were procured from Sangon (circ_0004904 WT, ARRDC3 3′UTR WT or circ_0004904 MUT, ARRDC3 3′UTR MUT).

### 2.9 RNA pull down assay

Bio-miR-19b-3p and Bio-miR-NC were from Sangon. HTR-8/SVneo cells were sonicated. The circ_0004904 probe was applied for cultivation with beads (Sigma) to produce probe-coated beads. Next cell lysates were incubated with circ_0004904 probe or oligo probe for 12 h. Then, the RNA stained with the beads was extracted for qRT-PCR.

### 2.10 Statistical assay

The tests were carried out in triplicate and the results were analyzed using SPSS 23.0 software (SPSS, USA). Student’s *t*-test and ANOVA were enforced to examine the differences. *P* < 0.05 was considered to be statistically significant.


**Ethics approval:** The present study was approved by the ethical review committee of The Affiliated Hospital of Hebei University. Written informed consent was obtained from all enrolled patients.
**Consent for publication:** Patients agree to participate in this work.

## 3 Results

### 3.1 Circ_0004904 was intensified in PE

**Figure 1:**
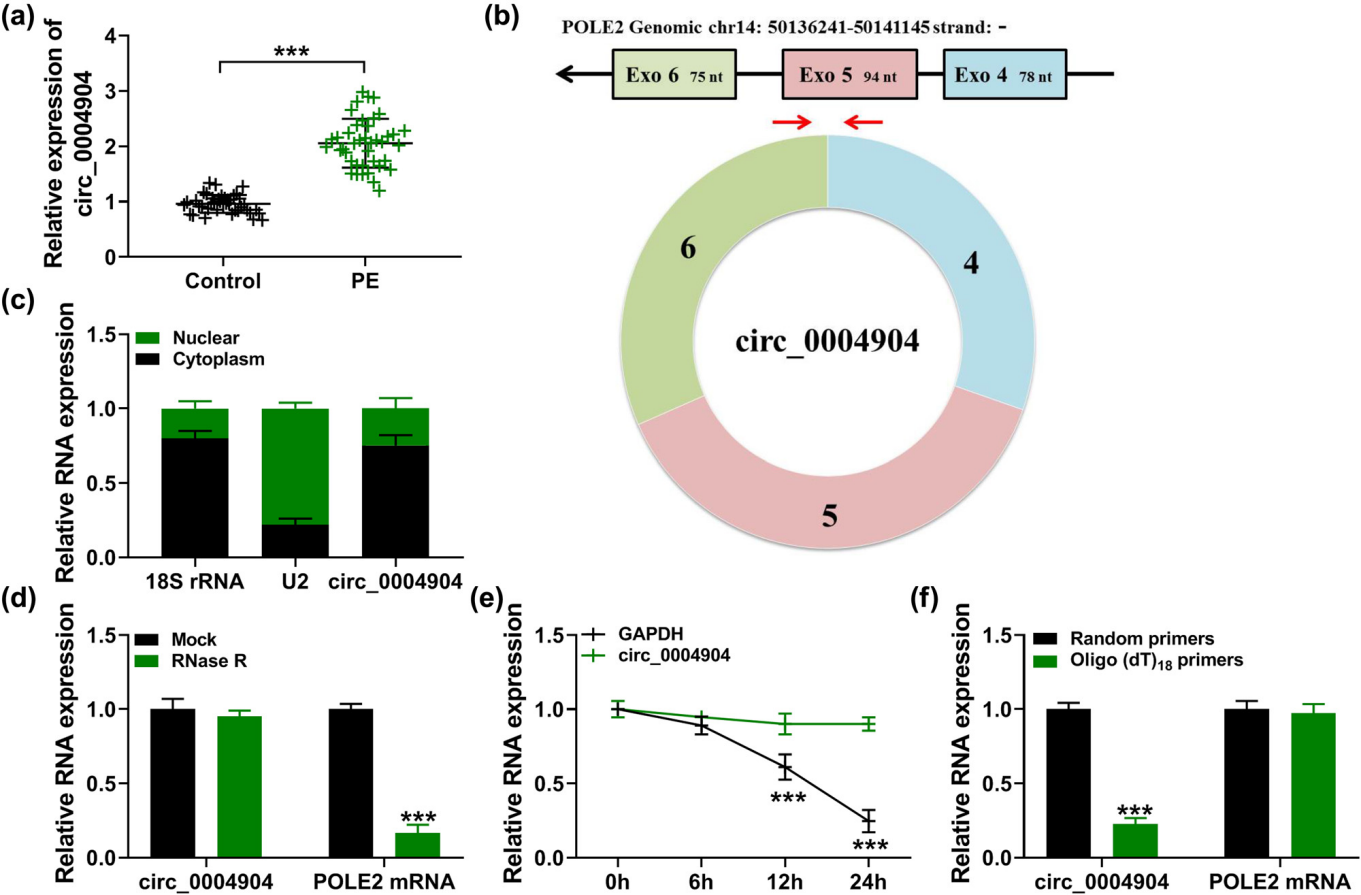
The relative level of circ_0004904 was enhanced in PE. (a) Relative levels of circ_0004904 in PE tissues and normal tissues were detected. (b) Location of circ_0004904 in genes and chromosomes. (c) Distribution of circ_0004904 in nucleus and cytoplasm. (d–f) Relative levels of circ_0004904 and POLE2 mRNA in HTR-8/SVneo cells. ***, *P* < 0.001.

Primarily, the outcomes displayed that in contrast to the normal tissues, circ_0004904 was intensified in PE placental tissues (Figure 1a). Circ_0004904 originates from the POLE2 gene and is located at chr14. Besides, circ_0004904 was formed by the exon 4–6 patchwork (Figure 1b). Moreover, the main distribution site of circ_0004904 was the cytoplasm (Figure 1c). Meanwhile, circ_0004904 content was not altered after RNase R or Act D treatment, while the POLE2 mRNA or GAPDH level was dropped (Figure 1d and e). In addition, circ_0004904 level was lower when Oligo (dT)18 primers were used compare with random primers, while the POLE2 mRNA was almost unchanged (Figure 1f). On the whole, these data demonstrated that circ_0004904 with a great steadiness structure was intensified in PE.

### 3.2 Circ_0004904 controlled trophoblast cell functions

**Figure 2:**
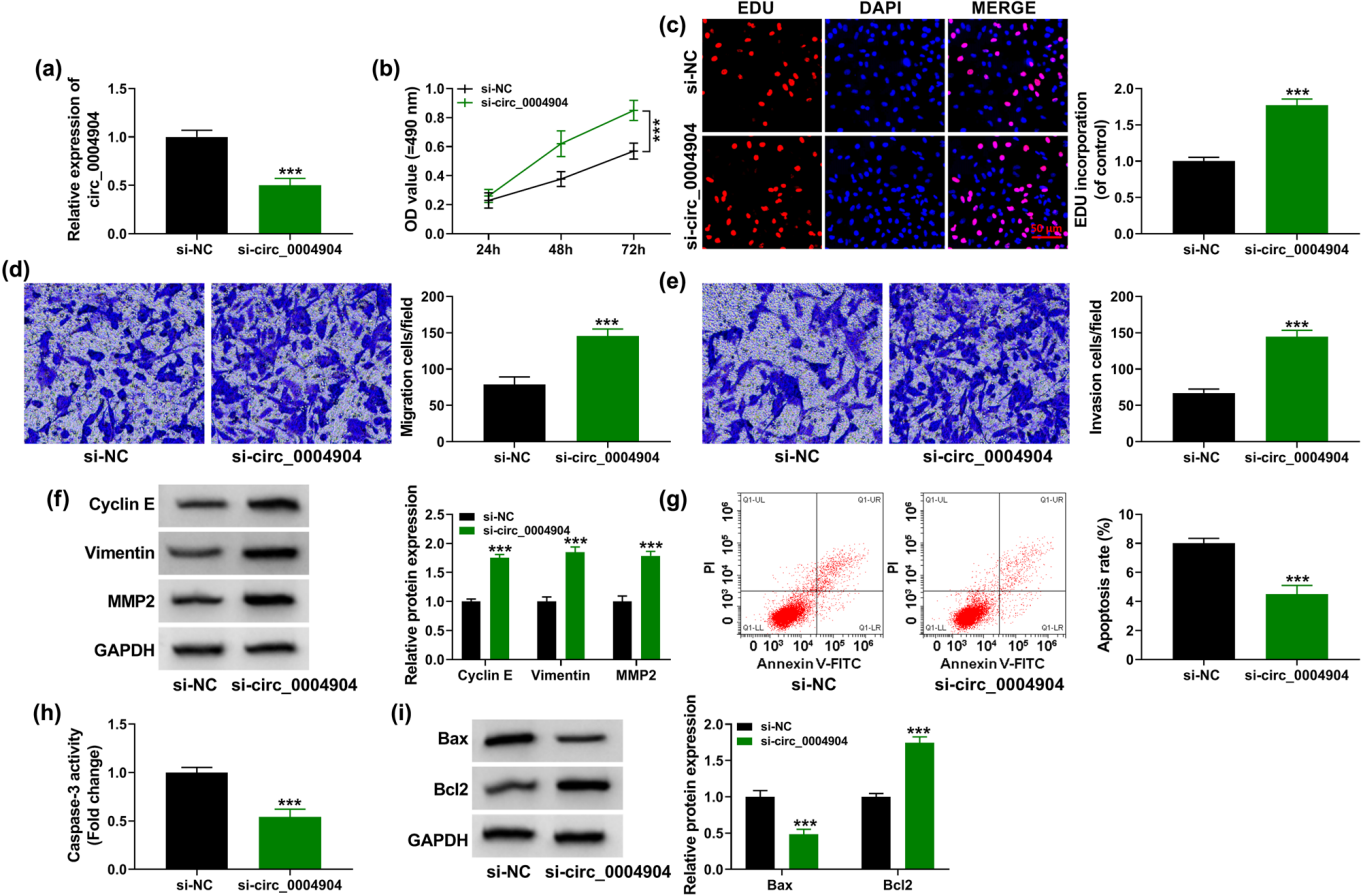
Circ_0004904 knockdown promoted HTR-8/SVneo cells development. HTR-8/SVneo cells were transfected with si-circ_0004904 or si-NC. (a) Knockdown competence of si-circ_0004904 was appraised by qRT-PCR. (b and c) MTT and EdU assays were enforced to evaluate cell proliferation. (d and e) Transwell assay was applied to assess cell migration and invasion. (f) Western blot assay was utilized to scrutinize the cyclin E, vimentin, and MMP2 contents. (g) Flow cytometry was conducted to assess the cell apoptosis. (h) The Caspase-3 activity was detected. (i) Western blot assay was utilized to scrutinize the Bax and Bcl2 contents. ***, *P* < 0.001.

The results exhibited that circ_0004904 expression was hampered in si-circ_0004904 group than in si-NC group (Figure 2a). Downregulated circ_0004904 encouraged cell proliferation in HTR-8/SVneo cells (Figure 2b and c). Moreover, silencing circ_0004904 expedited cell migration and invasion in HTR-8/SVneo cells (Figure 2d and e). Furthermore, cell proliferation, migration, and invasion -related proteins were detected. Circ_0004904 deficiency elevated the contents of cyclin E, vimentin, and MMP2 (Figure 2f). On the contrary, knockdown of circ_0004904 constrained cell apoptosis (Figure 2g). After that, the apoptosis-related proteins were measured, and the outcomes revealed that si-circ_0004904 transfection curbed the Caspase-3 activity and Bax levels, but elevated Bcl-2 expression (Figure 2h and i). These data exemplified that si-circ_0004904 was connected with the modulation of PE.

### 3.3 miR-19b-3p was targeted by circ_0004904

**Figure 3:**
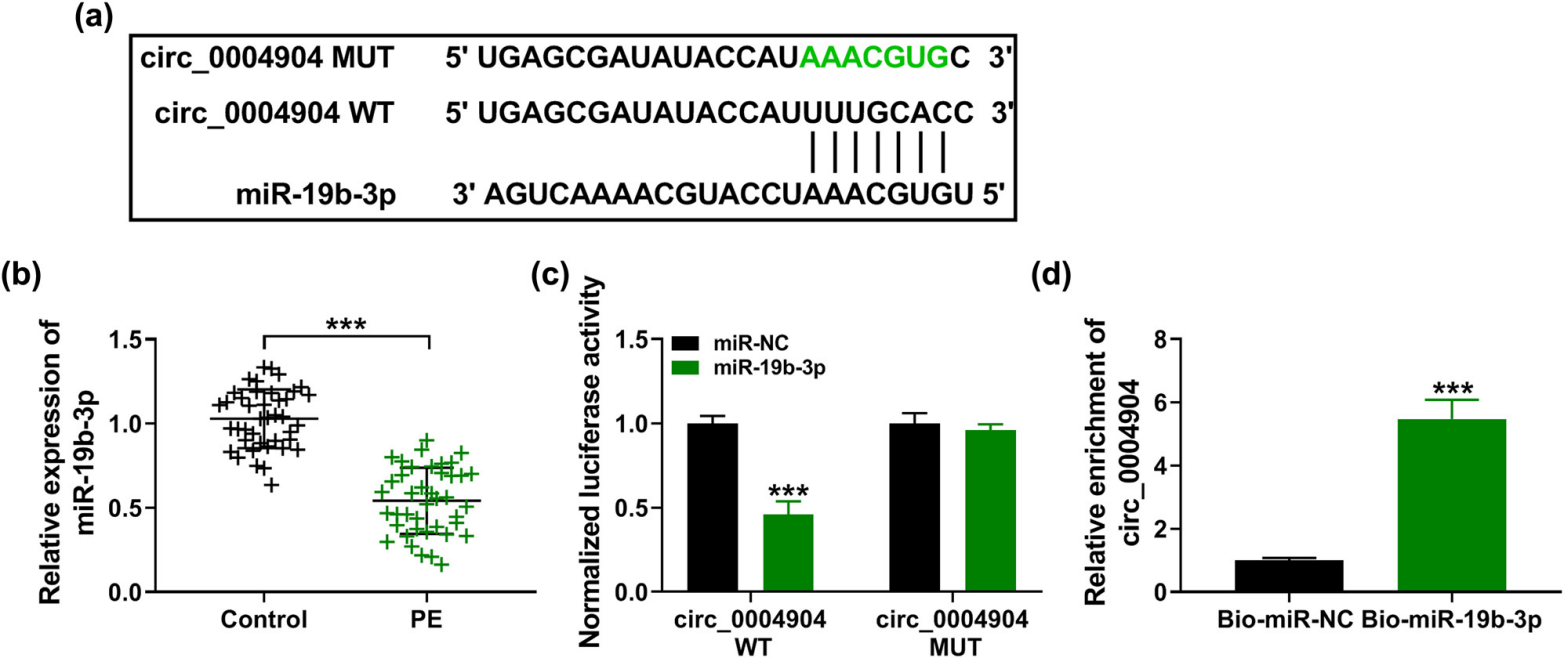
Interaction between miR-19b-3p and circ_0004904. (a) The complementary sites between miR-19b-3p and circ_0004904. (b) Relative levels of miR-19b-3p in PE tissues and normal tissues were detected. (c) HTR-8/SVneo cells were co-transfected with circ_0004904 WT or circ_0004904 MUT and miR-NC or miR-19b-3p, and the luciferase activity was detected. (d) Relative level of circ_0004904 was quantified by RNA pull down assay in HTR-8/SVneo cells. ***, *P* < 0.001.

The circ_0004904 included the bond sequences of miR-19b-3p, implying that miR-19b-3p might be a target of circ_0004904 (Figure 3a). Furthermore, with respect to the normal tissues, miR-19b-3p was decreased in PE placental tissues (Figure 3b). Subsequently, the luciferase activity of circ_0004904 WT in HTR-8/SVneo cells was lessened by miR-19b-3p, but there was no alteration in the circ_0004904 MUT group (Figure 3c). In addition, the targeted relationship of miR-19b-3p and circ_0004904 was also indicated by RNA pull down assay (Figure 3d). Collectively, these results proved that circ_0004904 repressed miR-19b-3p level by directly binding to miR-19b-3p.

### 3.4 The influence of circ_0004904 deficiency on cell functions was abrogated by miR-19b-3p inhibitor in trophoblast cells

**Figure 4:**
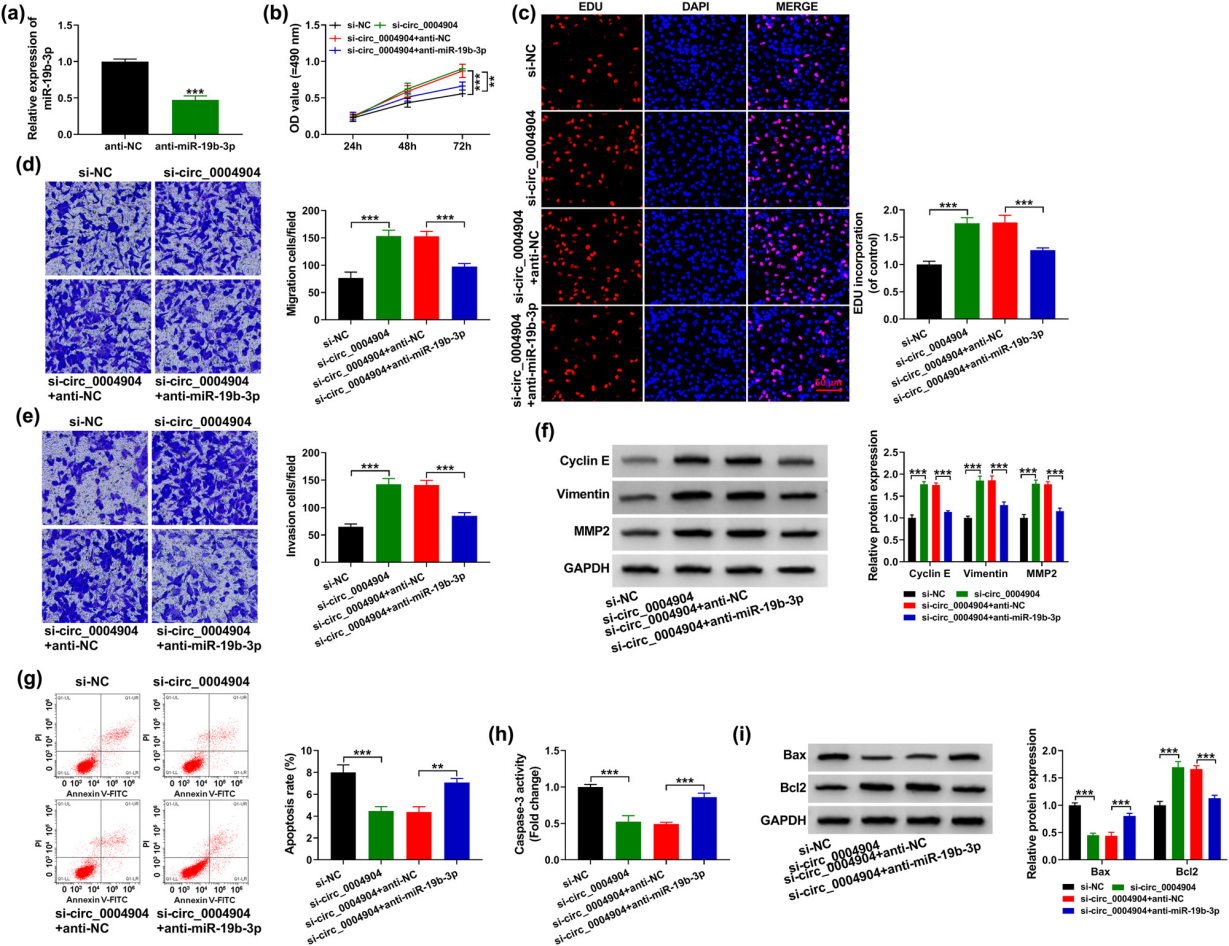
Circ_0004904 knockdown promoted HTR-8/SVneo cells development by regulating miR-19b-3p. (a) Knockdown competence of anti-miR-19b-3p was appraised by qRT-PCR. (b and c) MTT and EdU assays were enforced to evaluate cell proliferation. (d and e) Transwell assay was applied to assess cell migration and invasion. (f) Western blot assay was utilized to scrutinize the cyclin E, vimentin, and MMP2 contents. (g) Flow cytometry was conducted to assess the cell apoptosis. (h) The Caspase-3 activity was detected. (i) Western blot assay was utilized to scrutinize the Bax and Bcl2 contents. ***, *P* < 0.001.

The data unveiled that miR-19b-3p level was hampered in anti-miR-19b-3p group than in anti-NC group (Figure 4a). Then, the cell behaviors were detected. The data showed that reintroduction of anti-miR-19b-3p overturned the acceleration effect of circ_0004904 deficiency on cell proliferation in HTR-8/SVneo cells (Figure 4b and c). Moreover, anti-miR-19b-3p co-transfection abolished the promoting impacts of circ_0004904 knockdown on cell migration and invasion in HTR-8/SVneo cells (Figure 4d and e). Furthermore, downregulation of circ_0004904 elevated the contents of cyclin E, vimentin, and MMP2, while anti-miR-19b-3p could relieve the influence (Figure 4f). Additionally, knockdown of circ_0004904 constrained cell apoptosis; however, this impact was neutralized by anti-miR-19b-3p in HTR-8/SVneo cells (Figure 4g). Finally, anti-miR-19b-3p transfection counteracted the impact of si-circ_0004904 on the Caspase-3 activity, Bax, and Bcl-2 levels (Figure 4h and i). These outcomes disclosed that miR-19b-3p has a vital role in circ_0004904 deficiency-induced regulation in trophoblast cells.

### 3.5 ARRDC3 was a target of miR-19b-3p

**Figure 5:**
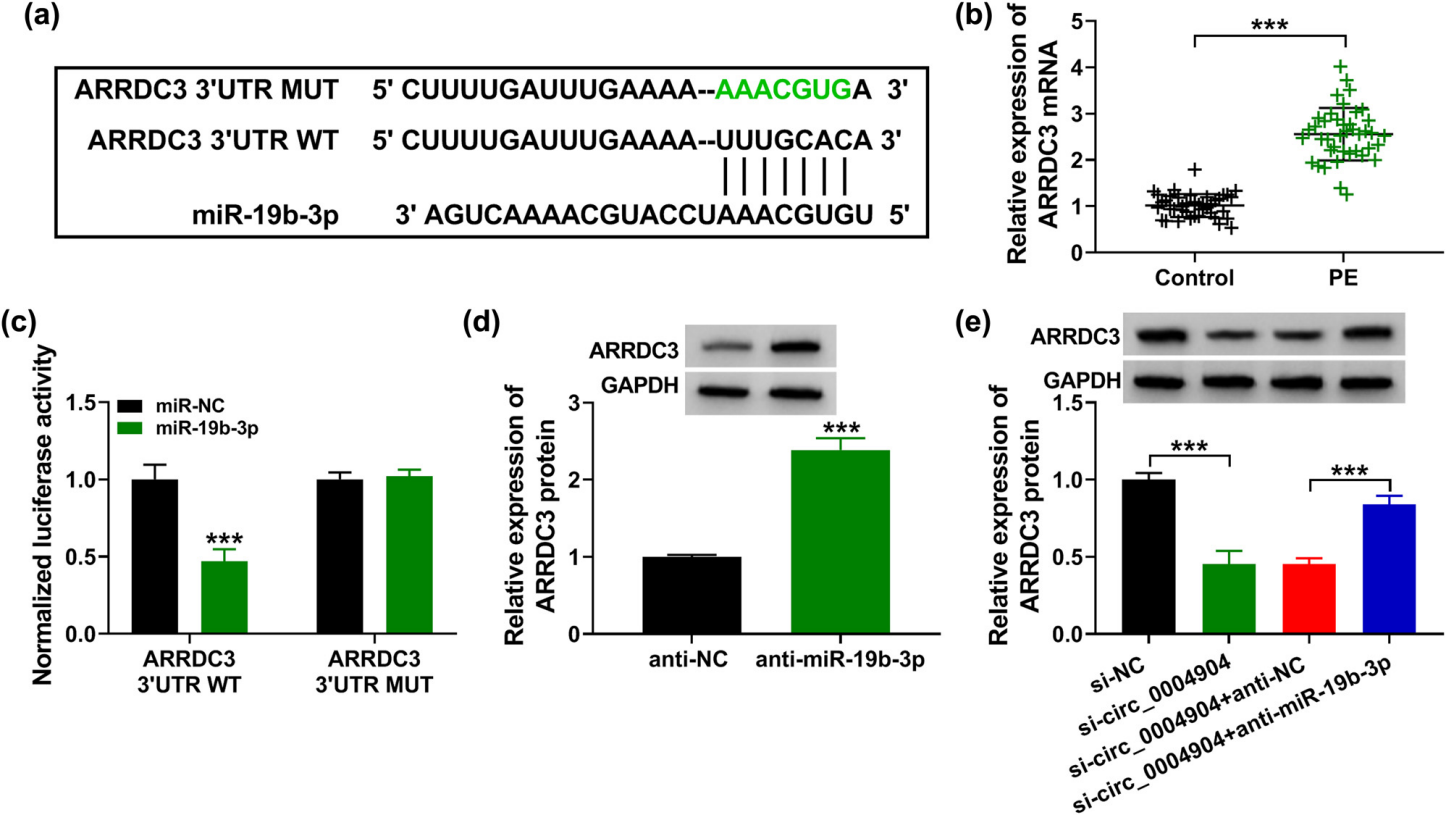
Interaction between miR-19b-3p and ARRDC3. (a) The complementary sites between miR-19b-3p and ARRDC3. (b) Relative levels of ARRDC3 in PE tissues and normal tissues were detected. (c) HTR-8/SVneo cells were co-transfected with ARRDC3 3′UTR WT or ARRDC3 3′UTR MUT and miR-NC or miR-19b-3p, and the luciferase activity was detected. (d and e) Relative levels of ARRDC3 in PE cells were quantified. ***, *P* < 0.001.

As presented in Figure 5a, starBase forecasted that ARRDC3 might be targeted by miR-19b-3p. Next relative to the normal tissues, ARRDC3 was enhanced in PE placental tissues (Figure 5b). Afterwards, the luciferase activity of ARRDC3 3′UTR WT in HTR-8/SVneo cells was lessened by miR-19b-3p, but there was no alteration in the ARRDC3 3′UTR MUT group (Figure 5c). Besides, anti-miR-19b-3p could enhance ARRDC3 content in HTR-8/SVneo cells (Figure 5d). Meanwhile, the ARRDC3 level was harmed by circ_0004904 deficiency, but elevated by anti-miR-19b-3p transfection (Figure 5e). These statistics disclosed that ARRDC3 was straightly targeted by miR-19b-3p in trophoblast cells.

### 3.6 Circ_0004904 regulated the trophoblast cell behaviors via ARRDC3

**Figure 6:**
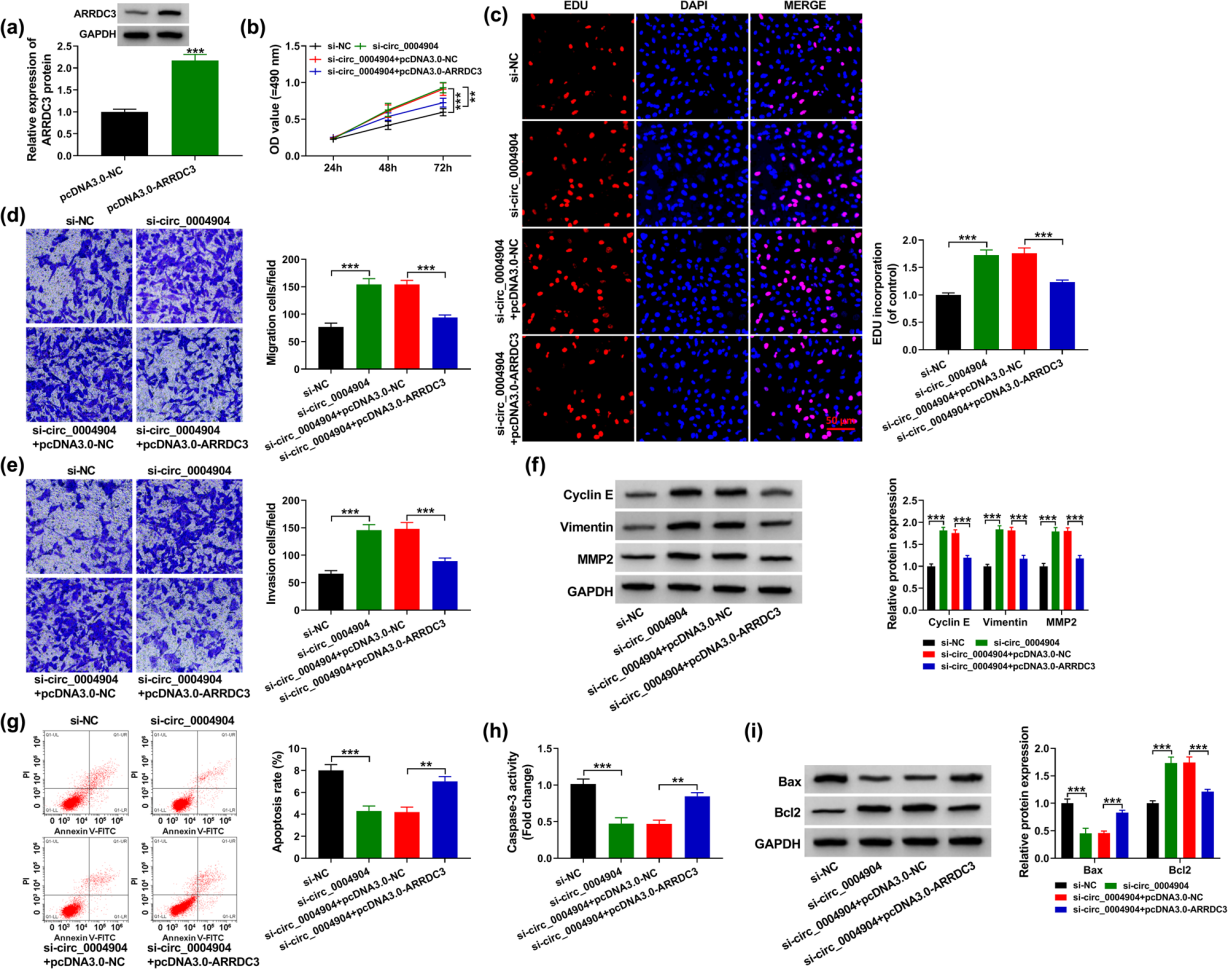
Circ_0004904 knockdown promoted HTR-8/SVneo cells development by regulating ARRDC3. (a) Upregulation competence of pcDNA3.0-ARRDC3 was appraised by qRT-PCR. (b and c) MTT and EdU assays were enforced to evaluate cell proliferation. (d and e) Transwell assay was applied to assess cell migration and invasion. (f) Western blot assay was utilized to scrutinize the cyclin E, vimentin, and MMP2 contents. (g) Flow cytometry was conducted to assess the cell apoptosis. (h) The Caspase-3 activity was detected. (i) Western blot assay was utilized to scrutinize the Bax and Bcl2 contents. ***, *P* < 0.001.

**Figure 7:**
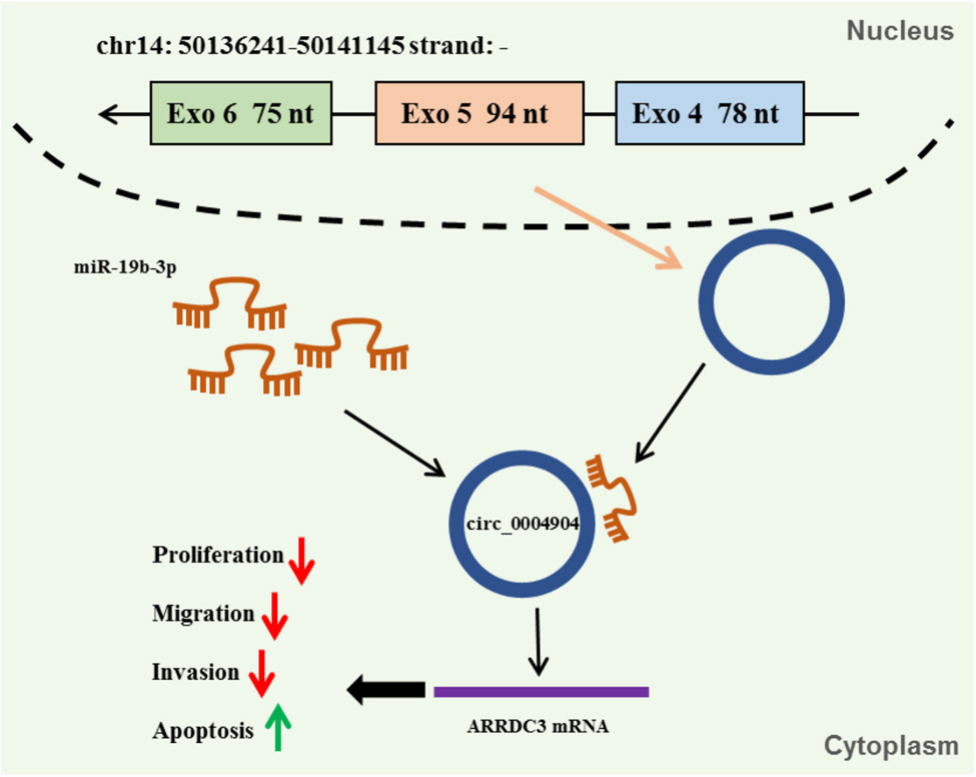
The regulation mechanism of circ_0004904/miR-19b-3p/ARRDC3.

The records exposed that ARRDC3 was upregulated in pcDNA3.0-ARRDC3 group than in pcDNA3.0-NC group (Figure 6a). The co-transfection of pcDNA3.0-ARRDC3 reversed the acceleration effect of circ_0004904 silencing on cell proliferation in HTR-8/SVneo cells (Figure 6b and c). Moreover, ARRDC3 overexpression abolished the elevation impacts of si-circ_0004904 on cell migration and invasion in HTR-8/SVneo cells (Figure 6d and e). Furthermore, downregulation of circ_0004904 elevated the contents of cyclin E, vimentin, and MMP2, while pcDNA3.0-ARRDC3 could relieve this influence (Figure 6f). In addition, silencing of circ_0004904 inhibited cell apoptosis, but this clogged impact was neutralized by ARRDC3 in HTR-8/SVneo cells (Figure 6g). Finally, ARRDC3 overexpression lessened the impact of si-circ_0004904 on the Caspase-3 activity, Bax, and Bcl-2 levels (Figure 6h and i). Furthermore, Figure 7 exhibited the regulation mechanism of circ_0004904/miR-19b-3p/ARRDC3 axis.

## 4 Discussion

According to the current results of clinical studies, it is not difficult to find that the treatment of PE is more difficult than prevention [25]. The symptoms of PE do not stop completely pathologically. Therefore, the current treatment approach is mainly aimed to prolong pregnancy by slowing down the pathological process [26,27]. In recent years, noninvasive methods, such as nucleic acids detection, have been proposed and developed for the early diagnosis of PE due to the release of molecules of the placental compartment [28,29]. Hence, we aimed to investigate the new molecules for PE treatment.

In accordance with this work, the circ_0004904 levels in PE tissues were remarkably elevated, which was similar to that reported by Jiang et al. [9]. These records speculated that circ_0004904 might play a vital role in PE. In this work, loss of function technique was applied to assess the underlying effects of circ_0004904 on trophoblast cells. The outcomes showed that circ_0004904 downregulation contributed to cell proliferation, migration, and invasion, but restrained cell apoptosis in trophoblast cells.

CircRNAs could act as miRNA sponge to control their biological role [13]. Previous studies have revealed that altered expression of some miRNAs plays important role in the onset and development of placental-induced diseases, such as fetal growth restriction [30] and PE [31]. Here the data displayed that circ_0004904 included the target sites of miR-19b-3p. Li et al. uncovered that miR-19b-3p inhibited osteoarthritis development [32]. Moreover, miR-19b-3p could regulate cell proliferation in human pancreatic cancer [33]. In this work, it was found that miR-19b-3p levels in PE tissues were remarkably restrained, which was similar to that described by Sandrim et al. [17]. Meanwhile, miR-19b-3p could target ARRDC3 to hamper PE development. Therefore, it was suggested that circ_0004904 exercised its function by the miR-19b-3p/ARRDC3 axis.

ARRDC3 has been shown to be involved in regulating the progression of many cancers, like colorectal cancer and breast cancer [19,20]. The information in this work presented that ARRDC3 level was abnormally intensified in PE, which was alike to Lei et al. description [21]. Moreover, ARRDC3 could promote trophoblast cell dysfunction.

In short, we presented that circ_0004904 acted as a sponge of miR-19b-3p to positively adjust ARRDC3, thus accelerating PE progression. These conclusions delivered a fresh train of thought for PE handling.
